# Body Mass Index Influence for the Personalization of the Monoclonal Antibodies Therapy for Psoriasis

**DOI:** 10.3390/life11121316

**Published:** 2021-11-29

**Authors:** Flavia Anghel, Diana Nitusca, Patricia Cristodor

**Affiliations:** 1 Department of Biochemistry and Pharmacology, “Victor Babe” University of Medicine and Pharmacy, Pta Eftimie Murgu Nr. 2, 300041 Timişoara, Romania; flaviaanghel7@gmail.com (F.A.); nitusca.diana@umft.ro (D.N.); 2 Center for Complex Networks Science, “Victor Babe” University of Medicine and Pharmacy, Pta Eftimie Murgu Nr. 2, 300041 Timişoara, Romania; 3 Department of Dermatology and Venerology, “Victor Babe” University of Medicine and Pharmacy, Pta Eftimie Murgu Nr. 2, 300041 Timişoara, Romania

**Keywords:** psoriasis, body mass index, monoclonal antibodies, drug-induced weight gain

## Abstract

Psoriasis is a chronic inflammatory, autoimmune-mediated disease that affects millions of individuals worldwide. Advances in treatment with biological agents represented by monoclonal antibodies, such as TNF-α inhibitors (TNFI), IL-17A and IL-12/23 antagonists have not only benefited from outstanding clinical efficacy with lower side effects compared to conventional systemic therapy, but also raised the standards towards therapeutic success, fact reflected in the greater Psoriasis Area and Severity Index (PASI) response rates. However, due to their relatively recent introduction in clinical practice, and despite their proven superior efficacy, further research is needed for monitoring the eventual changes in treatment-induced parameters, especially of metabolic origin. In this respect, initial reports stress on one particular comorbidity associated with psoriasis-obesity-which seems to be not only a risk and result of the disease, but also an adverse effect of long-term therapy with some biologics. The consequent drug-induced increase in body mass index (BMI) of patients suffering from psoriasis undergoing biological treatment appears to contribute to the progression of the disease, promote drug discontinuation and reduce overall clinical efficacy of monoclonal antibodies. Therefore, we review herein the impact of body weight (BMI) increase on the biological treatment of psoriasis, to further investigate on its relationship with the disease and aid on the management of treatment schemes that take into account individual characteristics of patients, such as body mass, for a more efficient and personalized therapy approach.

## 1. Introduction

Psoriasis is an autoimmune disease that induces chronic changes in the superficial layer of the skin (epidermis) by a faster development of the keratinocytes. This is a common, non-communicable disease that affects up to 125 million people worldwide, influenced by the genetics and epigenetics dissimilarities of the individual [1,2]. Morphologically, the skin appears to have an erythematous, scaly aspect, resulted from the overstimulation of the keratinocytes, angiogenesis with dilated blood vessels and the infiltration of T lymphocytes-mediated immune cells. The keratinocytes develop and proliferate in a faster (abnormal) way, with the maturation time shortening up to 3–5 days [3].

On the immunological level, studies show that a minor injury can cause the keratinocytes to stimulate the activation of the antigen-presenting dendritic cells leading to the primary trigger in psoriatic disease. The activated dendritic cells (DCs) secrete IL-12 and IL-23 (pro-inflammatory cytokines of the IL-12 family), essential for the activation and survival of Th1 and Th17 lymphocytes. Keratinocytes recruit DCs and IL-17-producing T cells via CCL20 chemokine attraction. DCs then drive the activation of T-cells and production of cytokines through IL-12 and IL-23. Furthermore, TNF-α is particularly linked to psoriatic lesions due to its ability to regulate the antigen-presenting quality of the DCs, which stimulates the infiltration of T-cells and activates several cytokines and chemokines, maintaining the lesions in a constant state of inflammation. In addition, this inflammation state is also fueled by chemokine attraction of neutrophils, driven by other cytokines like IL-17A (produced by Th17 cells), that regulate a faster proliferation with poor differentiation of keratinocytes [4].

For this rationale, there is a shifting trend in therapy towards the use of biological agents represented by monoclonal antibodies that neutralize these cytokines—TNF-α inhibitors (TNFI), such as infliximab, etanercept and adalimumab, IL-17A inhibitors (secukinumab and ixekizumab), as well as IL-12/23 antagonists, such as ustekinumab - that have surpassed conventional therapy for having greater efficacy and lower toxicity [5,6]. However, with few exceptions, current biologics are still under a fixed-dose therapy regimen that does not account for patients’ individual characteristics, which in turn may impede them from reaching higher clinical success [7].

The body mass index (BMI, kg/m^2^) of an individual is of particular interest, as high values appear to be both a risk and a result of the disease, and possibly a potential future predictor for treatment response. Recent research has shown that high BMI values negatively impact both disease progression and efficacy to biologics [8]. Therefore, considering BMI in clinical practice for personalizing therapy schemes with biologics could bring resourceful benefits in terms of drug pharmacokinetics, efficacy and ultimately prevent drug discontinuation. Therefore, the purpose of this report was to review available literature data on the influence of BMI upon the efficacy of biological treatment with different classes of monoclonal antibodies, and discuss the consequences for clinical practice.

## 2. Materials and Methods

### 2.1. Selection of Studies

We performed a review of available literature data that evaluated the effect and implication of body mass index/weight gain on the biological treatment with TNF-alpha inhibitors, IL-17A inhibitors and IL-12/23 inhibitors for psoriasis. Our aim was to investigate which monoclonal antibodies induce weight gain/body mass index increase following therapy and evaluate whether this effect will contribute to efficacy decrease.

All studies included in this review were collected by examining PubMed, Web of Knowledge, and DirectScience databases (up to 20th of September 2021), using the following terms: psoriasis, biological treatment, monoclonal antibodies, weight gain, body mass index, effect. These Medical Subject Headings (MeSH) terms were combined using “AND” and “OR” function with additional terms: TNF-α inhibitors, infliximab, etanercept, adalimumab, IL-17A inhibitors, secukinumab, ixekizumab, IL-12/23 inhibitors, ustekinumab. References from the selected articles were also screened in order to identify other potentially relevant studies. Regarding the search strategy period, included studies were published between 2000 and September 2021.

Duplicate references were excluded, after the electronic search in the aforementioned databases. The titles of the articles and the abstracts were examined based on the inclusion and exclusion criteria.

Two authors (D.N. and F.A.) retrieved supposedly eligible studies by examining titles, abstracts and full-text articles to be included in the present review. Disagreements were resolved by discussion and by consulting the third author (P.C.).

### 2.2. Inclusion and Exclusion Criteria

We selected research articles and controlled or observational clinical trials that contained data regarding treatment follow-up with monoclonal antibodies in patients diagnosed with psoriasis and the effect on body mass index and/or weight gain.

Inclusion criteria were: (1) original research articles or clinical trials including psoriasis patients treated with monoclonal antibodies and their effect on body mass index, (2) studies assessing the efficacy of biological treatment following body mass index increase, (3) drug-induced weight gain in relationship with PASI response rates, (4) articles published in English language.

Exclusion criteria were: (1) studies not performed on human subjects, (2) non-original studies, such as abstracts, letters to editors, and reviews, (3) duplicate reports, and (4) articles not written in English language.

Our study protocol was not prospectively registered.

### 2.3. Data Extraction

We selected from potentially eligible studies data regarding the number of patients included in the clinical controlled or observational trial, their age, the type (class) of monoclonal antibody administered for the treatment of psoriasis, PASI scores before and after treatment, weight or body mass index value following treatment with biologics, and efficacy of therapy following drug-induced weight gain. 

Due to the fact that the majority of the selected eligible studies were observational reports and did not contain sufficient data for a meta-analysis, the present report is a structured review of available data up to present.

### 2.4. End-Points

The primary end-point was the weight gain/body mass increase following therapy with some monoclonal antibodies (drug-induced weight gain) that can aid in personalization of the therapy for overweight/obese patients to shift towards biologics with neutral effect on weight.

The secondary end-point was the drug discontinuation pattern in cases of drug-induced weight gain. 

The third end-point was the reduced efficacy of biological treatment due to drug-induced weight gain, which in turn calls for pharmacokinetic evaluations and adjusting the dose based on the body mass index parameter. 

### 2.5. Study Characteristics 

We considered for our review only studies that presented data regarding the effect of monoclonal antibodies for psoriasis on the body mass index of patients with psoriasis. From the initial search, we found 133 articles relevant for our topic. 

**Figure 1:**
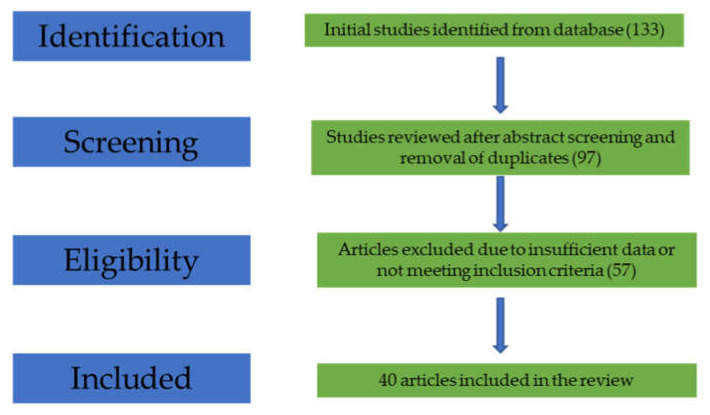
Flow-diagram of the study selection process.

After abstract screening, 97 reports were included (duplicate studies and non-original reports such as reviews and meta-analyses were removed). Following full-text reading, 57 articles were excluded due to lack of sufficient data for our interests, and 40 articles were included in this review (Figure 1).

## 3. Results

### 3.1. TNF-α Inhibitors (TNFI) and BMI

Over the years, studies have shown that pro-inflammatory cytokines (especially TNF-α) are excessively produced in mast cells of psoriatic plaques and in psoriatic skin lesions in general. For this rationale, the development of selective biologic agents that inhibit the production of TNF-α cytokine have proven to be real and optimized alternatives to the conventional therapies that have been used for decades, but with limited success [9].

Therefore, TNFI are among the most well-studied and widely used biologics for psoriasis therapy; however, recent reports show that they are also leading agents of drug-induced weight gain (BMI increase, Table 1) [10]. This in turn can be, at least partly explained based on the metabolic implications of TNF-α cytokine as a regulatory factor. Of note, lipolysis, suppression of growth factors, insulin and other anabolic hormones prove its involvement in overall weight decrease, and the inhibition of endogenous TNF-α therefore may lead to mass increase and accumulation of fat-free mass. This in turn will contribute to the undesired cardiovascular side effects of this class of drugs, and especially in matters of psoriasis, will negatively impact other comorbidities such as obesity, fact reflected in the drastic BMI increase [11,12]. 

In addition, it has been well studied over the years that patients suffering from psoriasis have a greater predisposition towards obesity (BMI > 30 kg/m2), and that overweight or obese psoriasis-free individuals have a 40% higher risk of developing late on-set psoriasis. This risk-result phenomenon could mainly arise due to the fact that both disorders have an immunological trigger, and similar molecular pathogenesis. Both psoriasis and obesity are linked to systemic inflammation that ultimately leads to an immunological response. In both disorders there is a higher TNF-α production from macrophages, and more recently the Psoriasis Area and Severity Index (PASI) score was positively correlated with BMI values [10]. The vicious cycle of weight gain appears to be fueled also by therapy with TNFI, based on their involvement in halting the regulatory roles of TNF-α cytokine in metabolic pathways involved in weight decrease.

**Table 1:** TNFI and the effect of weight gain (increased BMI) on drug efficacy.

TNFI	Structure and Mechanism	Effect on BMI/Weight Gain	Influence of BMI on Drug Efficacy	Commentary
**Infliximab**	mouse-derived chimeric IgG1; neutralizes both soluble, and membrane-bound TNF-α	Significant, gradual increase [8,10,13,14,15,16,17,18,19,20,21,22,23,24]	Increased BMI promotes drug discontinuation [11,12,26] Failing therapy [12] Steady decrease of PASI-75 with increasing BMI [21] Increased BMI leads to reduced efficacy and delayed response [27]	Weight-dependent dosing shows to increase efficacy [8,10]
**Etanercept**	prototypic recombinant fusion protein; inhibits only soluble TNF-α	Non-uniform [10,19] weight increase [16,17,18,23,24] Non-statistically significant weight increase [10,25] BMI increased more in subjects with normal weight at baseline [19]	Pharmacokinetic interactions due to wider adipose tissue [10,26] Drug discontinuation [11,12,26] BMI increase affects early clinical response [21]	Weight-dependent dosing not implemented to date [28]; should be taken in consideration to counteract pharmacokinetic issues Significant weight gain might call for the use of etanercept only in normal BMI individuals [8]
**Adalimumab**	phage display-derived, fully monoclonal antibody	Significant mass increase [10,18,19]	Pharmacokinetic interactions due to wider adipose tissue [10,26] Highest drug discontinuation [26] Strong diminished drug efficacy with BMI increase [8] No significant relationship between efficacy and body weight [22]	Conflicting studies call for further research to draw definitive conclusions

Therefore, the ultimate consequence of weight gain in psoriasis patients using TNFI is an overall tendency of drug discontinuation and/or reduced efficacy of the therapy. In this regard, Di Lernia et al. conducted a long-term observational, retrospective cohort study which included a minimum 1-year follow-up psoriasis patients undergoing treatment with TNFI and found for the first time that BMI is a promising predictor for drug discontinuation. Based on their statistical data regarding retention rates, the drug continuation percentages for all three drugs together (infliximab, etanercept and adalimumab) dropped from 67.43% at 12 months to 42.21% at 24 months [26]. In the same manner, another observational cohort study performed on Danish and Icelandic patients (data collected from the DANBIO and ICEBIO registries) suffering from psoriatic arthritis showed that obese patients were less compliant and had a shorter adherence to TNFI, with a median duration of the treatment of 2.5 years, significantly lower than non-obese individuals (5.9, *p* < 0.01), suggesting that obesity (BMI > 30 kg/m^2^) was a risk factor for drug discontinuation [11]. Yet another large-scale meta-analysis study encompassing 54 cohorts (total of 19372 patients) reported that an increase with only 1 kg/m^2^ in BMI had a 6,5% higher odds of therapy failure (OR = 1.065) in multiple immune-mediated inflammatory diseases including psoriasis and psoriatic arthritis, and obese patients had 60% higher odds of drug discontinuation (OR = 1.6) [12].

In addition, while all three TNFI contribute significantly to weight gain (BMI increase) and therefore to a drug discontinuation pattern in obese patients, studies have shown (but to a lesser extent that is worth researching more-in-depth), that differences in weight gain and treatment response occur between these biologic agents. Tan et al. showed that both infliximab and adalimumab therapies in psoriasis patients resulted in a significant weight increase after 12 weeks, which persisted also at week 24, but which was not statistically significant for etanercept [10]. It therefore appears that while infliximab and adalimumab show a continuous weight gain trend, by contrast, treatment with etanercept leads to a more non-uniform weight gain pattern. Namely, in the beginning of the therapy, the majority of patients experienced weight gain which gradually decreased in the long-term treatment (generally after 1 year of therapy). Interestingly, baseline normoponderol psoriatic patients experienced a more drastic BMI increase compared to overweight patients [19].

Furthermore, it appears that there are also manifold negative consequences of weight gain (BMI increase) regarding the actual efficacy of biological treatment with TNFI as well, in patients with continuous therapy. It is suggested that a higher BMI value leads to an overall lower PASI response rate. Namely, obese patients showed a lower improvement at 8 and 16 weeks, as only 29.1% of patients with BMI higher than 30 kg/m^2^ reached PASI75, compared to 41.7% of patients with BMI under 20 kg/m^2^ (week 8), and the trend persisted at week 16, in which the proportion of PASI75 achievement for normal weight patients was 59%, while for obese patients was 42.2% [21]. 

On the pharmacokinetic level, a fixed-dose therapy regimen lowers the efficacy in obese patients based on the affected clearance rate and expansion of the distribution volume caused by a wider adipose tissue. Therefore, the approach of weight-dependent dosing might benefit more in the upcoming future, as this personalized scheme has been proven effective for infliximab in 95–100 kg individuals [8,10]. It has been shown that weight-adjusted dosing contributes significantly to the achievement of similar PASI75 response rates, regardless of BMI values (PASI75 rates were 77.5%, 78.3% and 74.4% for normal, overweight and obese patients when infliximab was weight-adjusted for) [29].

Nonetheless, for adalimumab, debate is still undergoing, since a research report showed that weight increase does not affect treatment efficacy in a significant manner [22], while others showed that in fact this drug contributes to the most significant mass increase, highest drug discontinuation and a strong negative influence on pharmacological efficacy in relationship with BMI increase [8,10,26].

### 3.2. IL-17A Inhibitors and BMI

It is well-known that IL-17 cytokine family (produced by Th17 cells) is involved in inflammatory autoimmune-mediated diseases such as psoriasis, where tissue inflammation also occurs due to high expression of IL-17A and IL-17F. These cytokines lead to both neutrophil accumulation, formation of skin lesions and upregulation of chemokines involved in the pathophysiology of psoriasis [30]. On that account, the development of biologic agents represented by monoclonal antibodies that target and inhibit the expression of IL-17A have proven to grant outstanding clinical efficacy in the treatment of psoriasis. Thus, secukinumab and ixekizumab show that, in general, PASI75 was achieved in more than 80% of the patients treated with IL-17A antagonists [31,32]. Furthermore, evidence from multiple clinical trials suggests that IL-17A inhibitors were superior in efficacy compared to other biologics such as TNFI and anti-IL-12/23. For subcutaneous treatment with secukinumab, more than 80% of patients achieved a PASI90 response rate, setting therefore a new higher standard [31,33,34]. 

However, owing to their recent introduction in clinical practice, limited information is available regarding IL-17A inhibitors effect on weight gain, which requires extensive research. In this respect, an initial, comparative research study conducted by Takamura et al. (2018) showed for the first time that treatment with secukinumab does not affect body weight and reported constant BMI levels after 7 months of therapy. Their 7-year retrospective study showed that, compared to infliximab (where mean BMI increased from 24.7 to 25.7 kg/m^2^ after 7 months), treatment with secukinumab maintained mean BMI levels to a constant value of 25.2. Based on their findings, it is therefore suggested that secukinumab exerts a neutral effect on body weight and could therefore constitute a more advantageous alternative for obese patients undergoing TNFI therapy [35]. In the same manner, results from a 12-week phase 3 clinical trial (UNCOVER-1, UNCOVER-2, and UNCOVER-3) measuring for cardiovascular and other parameters showed that ixekizumab had a neutral effect on both cardiovascular-related variables and body mass, as no weight gain was observed in patients receiving ixekizumab therapy at 12-week follow-up treatment [36]. Another report conducted by Reich et al. studying the body weight effect on the response to ixekizumab showed that both efficacy and safety profiles were similar irrespective to body weight of patients. In addition, no significant differences in PASI 75 response rates were observed across bodyweight categories [37]. Yet another recent report showed that treatment with IL-17A inhibitors in severe psoriasis did not significantly change body composition parameters such as body mass (median baseline BMI was constant at 32.8 kg/m^2^) [38]. Table 2 presents the general characteristics and main literature findings on IL-17A inhibitors in relationship with BMI.

### 3.3. IL-12/23 Inhibitors and BMI

Ustekinumab is the first-in-class anti-12/23p40 monoclonal antibody which has proven exceptional efficacy with low immunogenicity that translated into substantial therapeutic benefit for psoriasis patients in the last decade. It targets two cytokines (IL-12 and IL-23) known for their involvement in the pathogenesis of this dermatological disease [39]. However, when investigated in relationship with BMI and mass increase, it appears that ustekinumab is situated in the middle-way compared to TNFI and to IL-17A inhibitors, for that treatment does not produce any BMI modifications or other metabolic disturbances, safety and tolerance is increased when compared to TNFI but efficacy is reduced in obese patients, relative to IL-17A inhibitors for which available yet limited data showed comparable efficacy regardless of BMI [40].

Therefore, the neutral effect on body weight makes ustekinumab a better alternative for obese patients, with few important aspects to be taken into account regarding efficacy. Pharmacokinetics reports have shown that bodyweight is an important variable affecting distribution volume and clearance of ustekinumab. PHOENIX1 and PHOENIX2 studies have noted a proportional decrease in serum concentration with mass increase, being even undetectable in 120 and 130 kg patients [41]. In addition, long-term extensions of PHOENIX clinical trials showed a decreased efficacy of ustekinumab in patients with higher BMI values, and a decrease in PASI response rates in individuals with BMI > 25 kg/m^2^ [42,43,44]. Thus, phase 3 clinical trials evaluated the potential weight-adjusted dosing strategy regarding efficacy optimization and found out that, by doubling the dose from 45 mg to 90 mg, patients weighing over 100 kg benefited from an increased efficacy (PASI90 was 20% higher in the 90-mg group, *p* < 0.0001). Another study comparing efficacy between etanercept and ustekinumab (ACCEPT trial) showed similar weight-related differences, with PASI scores significantly lower in individuals weighing > 100 kg [45]. Subsequently, therapy failure of insufficient treatment response will affect compliance and adherence to therapy, leading to drug discontinuation, as seen in other classes of biologics, where BMI could potentially constitute a predictor for treatment termination, as suggested by the BioCAPTURE registry evaluating patients undergoing ustekinumab treatment [46]. Nonetheless, as individual weight adjusted dosing has provided better and positive treatment responses, EMEA reflected this particularity in the Summary of Product Characteristics, where it is suggested to double the dose to 90 mg in over 100 kg patients, advancing therefore towards a more personalized therapy approach [47]. Main findings on ustekinumab and its association with BMI can be found in Table 3.

**Table 2:** IL-17A and the effect of weight gain (increased BMI) on drug efficacy.

IL-17A Inhibitors	Structure and Mechanism	Effect on BMI/Weight Gain	Influence of BMI on Drug Efficacy	Commentary
**Secukinumab**	recombinant fully human IgG1/kappa monoclonal antibody	Constant BMI levels; no weight gain [35,38]	Similar drug efficacy regardless of BMI values [35]; one study revealed that individuals <90kg had higher response rates [48]	Could be a better alternative for obese patients; Lack of sufficient data requires for extensive research to draw definitive conclusions
**Ixekizumab**	complete monoclonal antibody of the subclass IgG4	No significant weight gain [36,38]; Lack of sufficient data	Similar drug efficacy regardless of BMI values [37]	Could be a better alternative for obese patients; Lack of sufficient data requires for extensive research to draw definitive conclusions

**Table 3:** IL-12/23 and the effect of weight gain (increased BMI) on drug efficacy.

IL-12/23 Inhibitors	Structure and Mechanism	Effect on BMI/Weight Gain	Influence of BMI on Drug Efficacy	Commentary
**Ustekinumab**	fully human monoclonal antibody (mAb) that binds specifically to IL-12/IL-23p40	No reported BMI changes following treatment [40]	Pharmacokinetic interactions affecting serum concentration and drug clearance [41] Reduced PASI response rates (efficacy) with mass increase [42,43,44,45]	Could be a better alternative for obese patients; Doubling the dose in individuals weighing more than 100 kg significantly increased efficacy [45,46,47]

**Figure 2:**
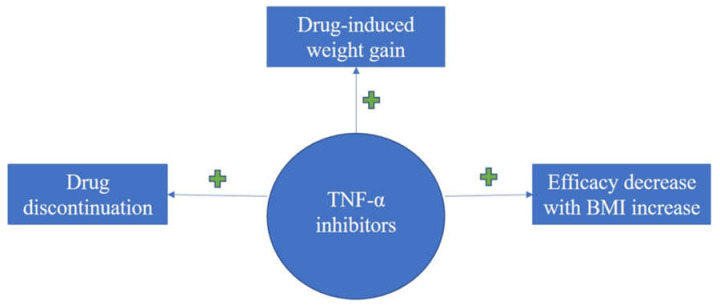
Diagram on evidence regarding BMI impact upon treatment response with TNFI for psoriasis; TNFI induces drug discontinuation, weight gain and the efficacy is decreased with increase in BMI values.

**Figure 3:**
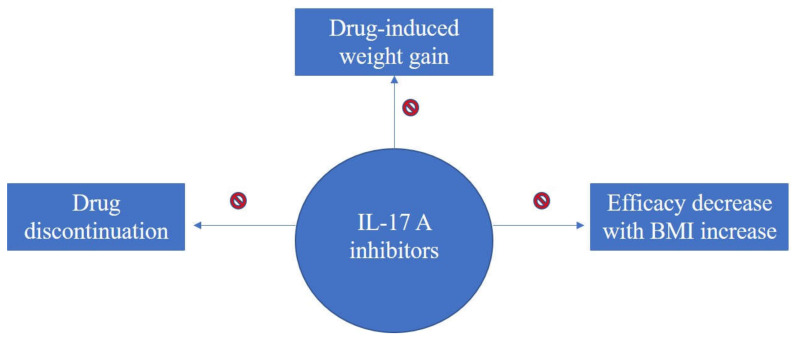
Diagram on evidence regarding BMI impact upon treatment response with IL-17A inhibitors for psoriasis; IL-17A inhibitors have neutral effect on weight gain and no decrease in efficacy has been reported for increasing BMI.

**Figure 4:**
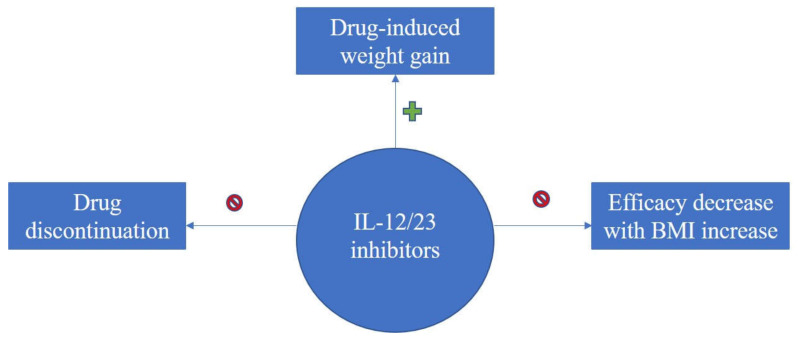
Diagram on evidence regarding BMI impact upon treatment response with IL-12/23 inhibitors for psoriasis; IL-12/23 inhibitors induce weight gain, however no drug discontinuation or decrease in efficacy with BMI increase was reported.

Taken together, an overview of literature findings regarding therapy-induced weight increase, consequences upon efficacy and overall treatment response of biologics in association with BMI for the treatment of psoriasis can be seen in the flow-diagram of Figure 2, Figure 3 and Figure 4.

## 4. Discussion and Future Perspectives

Since weight gain and obesity appear to be both a risk factor and a result of developing psoriasis, which can convey multiple therapeutic disadvantages (especially of pharmacokinetic nature), one effective measure of achieving minimal disease activity and improve treatment response with biologics seems to be a successful weight reduction, even regardless of the type of diet [49]. It is therefore of great importance in clinical practice that psoriasis patients are strongly encouraged by practicians to undergo different types of weight loss interventions, depending on the severity of each individual’s overweight or obese status. For mild to moderate cases, performing physical exercise, adhering to low-calorie diet and adopting a generally healthy lifestyle that includes smoking cessation, reducing alcohol consumption and avoiding fat diet seem to be adjuvant aids in conjunction with pharmacological therapy, leading overall to a significant improvement of PASI scores [50]. For severe cases of obesity, gastric bypass surgery is being proposed as an effective, yet disputable strategy to overcome disease exacerbation and improve treatment efficacy [51]. Moreover, weight reduction is definitely a paramount pillar for the cutback of not only psoriasis-related symptomatology and severity related to inflammation and cellular proliferation, but also for the improvement of metabolic syndrome and other comorbidities of cardiovascular origin as well [52].

Nevertheless, while weight control plays an important role in both disease prevention and progression, evidence strongly suggests that BMI decrease has a great impact on treatment response and efficacy of biologics, especially on agents subjected to fixed-dose therapy schemes. Clinical observational reports as well as controlled trials draw attention to the heterogeneity of treatment response with biologics and point out that BMI value is a key factor for efficacy and improvement of PASI scores, as seen particularly in the case of etanercept [53,54]. To overcome reduced treatment response in obese patients, some biologics have already been subjected to successful weight-adjusted dosing schemes, as aforementioned (infliximab, ustekinumab). It is therefore suggested that other biologics, especially from TNFI (etanercept, adalimumab) class for which evidence reports reduced efficacy with increasing BMI values might benefit from ameliorated drug response if dose will be adjusted based on bodyweight. Hence, this in turn calls for pharmacoeconomics studies and further investigation of subpopulations of patients who would obtain maximum benefit from weight-adjusted schemes. Increasing the dose in overweight and obese patients could provide a better treatment response in other biologics affected by BMI values; however, the economical and ethical implications have to be taken into consideration as well.

In addition, our results show that TNFI are the key players involved in drug-induced weight gain, relative to the other classes of biologics investigated. As possible mechanisms, it appears that TNF-α could have both central and peripheric anorexigenic effects [13]. On one hand, TNF-α induces appetite loss by hypothalamic signaling at the arcuate nucleus level, via the stimulation of the activation and release of anorexigenic neuropeptides (corticotropin-releasing factor), and via the inhibition of orexigenic neuropeptide Y (NPY) signaling network. It has been shown that TNF-α stimulates the AMP-activated protein kinase (AMPK) signaling pathway, which is involved in appetite and weight loss [55]. On the other hand, TNF-α appears to contribute to weight loss at the peripheric level, by promoting protein catabolism in mature muscle cells, via the activation of nuclear factor (NF)-κB and upregulation of the ubiquitin/proteasome pathway [56]. Therefore, by inhibiting TNF-α, both central and peripheric anorexigenic effects of this cytokine could be counteracted. However, some degree of inconsistency still exists, since other studies have reported the involvement of TNF-α in insulin resistance and obesity. Hence, given its pleiotropic nature, additional research is warranted to establish whether the roles and actions of TNF-α cytokine are specific to dose and/or context [57].

Taken together, considering that up-to-date available reports reveal a neutral effect of IL-17A inhibitors upon body weight and BMI values, our review might suggest the replacement of TNFI in overweight or obese patients with anti-IL17A such as secukinumab and ixekizumab, which show no weight gain trend following therapy and preserved their efficacy regardless of BMI values as well.

To conclude, BMI emerges as an important therapy-modifying factor, and further research is warranted in order to better determine its potential predictor role in biological treatment efficacy. Among metabolic parameters considered for clinical trials, it appears that BMI is of particular importance and it is suggested to be studied more-in-depth as an independent stratification parameter to evaluate its therapeutic prognostic future. Moreover, recent biologics included in clinical use such as secukinumab and ixekizumab from anti-IL17A family are still in need for further investigation as initial reports show neutral effect in both drug-induced weight gain and BMI-related treatment response, and therefore the novel era of biological agents holds great hope for optimized therapy outcomes in matters of psoriasis.

## Data Availability

All data is available in the manuscript.

## References

[1] Nestle F.O., Kaplan D.H., Barker J. (2009). Psoriasis. New Engl. J. Med..

[2] Parisi R., Symmons D.P., Griffiths C.E., Ashcroft D.M. (2013). Global epidemiology of psoriasis: A systematic review of incidence and prevalence. J. Investig. Dermatol..

[3] Ogawa K., Okada Y. (2020). The current landscape of psoriasis genetics in 2020. J. Dermatol. Sci..

[4] Lowes M.A., Suárez-Fariñas M., Krueger J.G. (2014). Immunology of psoriasis. Annu. Rev. Immunol..

[5] Brownstone N.D., Hong J., Mosca M., Hadeler E., Liao W., Bhutani T., Koo J. (2021). Biologic treatments of psoriasis: An update for the Clinician. Biologics.

[6] Rønholt K., Iversen L. (2017). Old and new biological therapies for psoriasis. Int J. Mol. Sci..

[7] Bahner J.D., Cao L.Y., Korman N.J. (2009). Biologics in the management of psoriasis. Clin. Cosmet. Investig. Dermatol..

[8] Puig L. (2011). Obesity and psoriasis: Body weight and body mass index influence the response to biological treatment. J. Eur. Acad. Dermatol. Venereol..

[9] Tobin A.M., Kirby B. (2005). TNF alpha inhibitors in the treatment of psoriasis and psoriatic arthritis. BioDrugs.

[10] Tan E., Baker C., Foley P. (2013). Weight gain and tumour necrosis factor-alpha inhibitors in patients with psoriasis. Austral. J. Dermatol..

[11] Højgaard P., Glintborg B., Kristensen L.E., Gudbjornsson B., Love T.J., Dreyer L. (2016). The influence of obesity on response to tumour necrosis factor-α inhibitors in psoriatic arthritis: Results from the DANBIO and ICEBIO registries. Rheumatology.

[12] Singh S., Facciorusso A., Singh A.G., Casteele N.V., Zarrinpar A., Prokop L.J., Grunvald E.L., Curtis J.R., Sandborn W.J. (2018). Obesity and response to anti-tumor necrosis factor-α agents in patients with select immune-mediated inflammatory diseases: A systematic review and meta-analysis. PLoS ONE.

[13] Patsalos O., Dalton B., Leppanen J., Ibrahim M., Himmerich H. (2020). Impact of TNF-α Inhibitors on Body Weight and BMI: A Systematic Review and Meta-Analysis. Front. Pharmacol..

[14] Florin V., Cottencin A.C., Delaporte E., Staumont-Sallé D. (2013). Body weight increment in patients treated with infliximab for plaque psoriasis. J. Eur. Acad. Dermatol. Venereol..

[15] Mahé E., Reguiai Z., Barthelemy H., Quiles-Tsimaratos N., Chaby G., Girard C., Estève E., Maccari F., Descamps V., Schmutz J.L. (2014). Evaluation of risk factors for body weight increment in psoriatic patients on infliximab: A multicentre, cross-sectional study. J. Eur. Acad. Dermatol. Venereol..

[16] Prignano F., Ricceri F., Pescitelli L., Buggiani G., Troiano M., Zanieri F., Rossari S., Lotti T. (2009). Comparison of body weight and clinical-parameter changes following the treatment of plaque psoriasis with biological therapies. Curr. Med. Res. Op..

[17] Gisondi P., Cotena C., Tessari G., Girolomoni G. (2008). Anti-tumour necrosis factor-alpha therapy increases body weight in patients with chronic plaque psoriasis: A retrospective cohort study. J. Eur. Acad. Dermatol. Venereol..

[18] Onsun N., Akaslan T.Ç., Sallahoglu K., Gülcan A.S., Bulut H., Yabacı A. (2021). Effects of TNF inhibitors and an IL12/23 inhibitor on changes in body weight and adipokine levels in psoriasis patients: A 48-week comparative study. J. Derm. Treat..

[19] Saraceno R., Schipani C., Mazzotta A., Esposito M., Di Renzo L., De Lorenzo A., Chimenti S. (2008). Effect of anti-tumor necrosis factor-alpha therapies on body mass index in patients with psoriasis. Pharm. Res..

[20] Takamura S., Takahashi A., Inoue Y., Teraki Y. (2018). Effects of tumor necrosis factor-α, interleukin-23 and interleukin-17A inhibitors on bodyweight and body mass index in patients with psoriasis. J. Dermatol..

[21] Naldi L., Addis A., Chimenti S., Giannetti A., Picardo M., Tomino C., Maccarone M., Chatenoud L., Bertuccio P., Caggese E. (2008). Impact of body mass index and obesity on clinical response to systemic treatment for psoriasis: Evidence from the Psocare Project. Dermatology.

[22] Chiricozzi A., Zangrilli A., Bavetta M., Bianchi L., Chimenti S., Saraceno R. (2017). Real-life 9-year experience with adalimumab in psoriasis and psoriatic arthritis: Results of a single-centre, retrospective study. J. Eur. Acad. Dermatol. Venereol..

[23] Esposito M., Mazzotta A., Saraceno R., Schipani C., Chimenti S. (2009). Influence and variation of the body mass index in patients treated with etanercept for plaque-type psoriasis. Int J. Immunopat. Pharm..

[24] Renzo L.D., Saraceno R., Schipani C., Rizzo M., Bianchi A., Noce A., Esposito M., Tiberti S., Chimenti S., De Lorenzo A. (2011). Prospective assessment of body weight and body composition changes in patients with psoriasis receiving anti-TNF-α treatment. Dermatol. Ther..

[25] Ross C., Marshman G., Grillo M., Stanford T. (2016). Biological therapies for psoriasis: Adherence and outcome analysis from a clinical perspective. Australas. J. Derm..

[26] Di Lernia V., Tasin L., Pellicano R., Zumiani G., Albertini G. (2012). Impact of body mass index on retention rates of anti-TNF-alfa drugs in daily practice for psoriasis. J. Dermatol. Treat..

[27] Duarte A.A. (2011). Moderate to severe psoriasis treated with infliximab—53 patients: Patients profile, efficacy and adverse effects. Ann. Bras. Dermatol..

[28] Nguyen T.U., Koo J. (2009). Etanercept in the treatment of plaque psoriasis. Clin. Cosmet. Investig. Dermatol..

[29] Reich K., Gottlieb A.B., Kimball A., Li S. (2006). Consistency of infliximab response across subgroups of patients with psoriasis: Integrated results from randomized controlled clinical trials. J. Am. Acad. Dermatol..

[30] Von Stebut E., Boehncke W.H., Ghoreschi K., Gori T., Kaya Z., Thaci D., Schäffler A. (2020). IL-17A in psoriasis and beyond: Cardiovascular and metabolic implications. Front. Immunol..

[31] Langley R.G., Elewski B.E., Lebwohl M., Reich K., Griffiths C.E., Papp K.A., Puig L., Nakagawa H., Spelman L., Sigurgeirsson B. (2014). Secukinumab in plaque psoriasis—Results of two phase 3 trials. New Engl. J. Med..

[32] Gordon K.B., Colombel J.F., Hardin D.S. (2016). Phase 3 trials of ixekizumab in moderate-to-severe plaque psoriasis. New Engl. J. Med..

[33] Thaçi D., Blauvelt A., Reich K., Tsai T.-F., Vanaclocha F., Kingo K., Ziv M., Pinter A., Hugot S., You R. (2015). Secukinumab is superior to ustekinumab in clearing skin of subjects with moderate to severe plaque psoriasis: CLEAR, a randomized controlled trial. J. Am. Acad. Dermatol..

[34] Blauvelt A., Reich K., Tsai T.F., Tyring S., Vanaclocha F., Kingo K., Ziv M., Pinter A., Vender R., Hugot S. (2017). Secukinumab is superior to ustekinumab in clearing skin of subjects with moderate-to-severe plaque psoriasis up to 1 year: Results from the CLEAR study. J. Am. Acad. Dermatol..

[35] Pirro F., Caldarola G., Chiricozzi A., Burlando M., Mariani M., Parodi A., Peris K., De Simone C. (2021). Impact of Body Mass Index on the Efficacy of Biological Therapies in Patients with Psoriasis: A Real-World Study. Clin Drug Investig..

[36] Egeberg A., Wu J.J., Korman N., Solomon J.A., Goldblum O., Zhao F., Mallbris L. (2018). Ixekizumab treatment shows a neutral impact on cardiovascular parameters in patients with moderate-to-severe plaque psoriasis: Results from UNCOVER-1, UNCOVER-2, and UNCOVER-3. J. Am. Acad. Dermatol..

[37] Reich K., Puig L., Mallbris L., Zhang L., Osuntokun O., Leonardi C. (2017). The effect of bodyweight on the efficacy and safety of ixekizumab: Results from an integrated database of three randomised, controlled Phase 3 studies of patients with moderate-to-severe plaque psoriasis. J. Eur. Acad. Dermatol. Venereol..

[38] Piros É.A., Szabó Á., Rencz F., Brodszky V., Wikonkál N., Miheller P., Horváth M., Holló P. (2021). Anti-interleukin-17 therapy of severe psoriatic patients results in an improvement of serum lipid and inflammatory parameters’ levels, but has no effect on body composition parameters. Life.

[39] Yeilding N., Szapary P., Brodmerkel C., Benson J., Plotnick M., Zhou H., Goyal K., Schenkel B., Giles-Komar J., Mascelli M.A. (2011). Development of the IL-12/23 antagonist ustekinumab in psoriasis: Past, present, and future perspectives. Ann. N. Y. Acad. Sci..

[40] Gisondi P., Conti A., Galdo G., Piaserico S., De Simone C., Girolomoni G. (2013). Ustekinumab does not increase body mass index in patients with chronic plaque psoriasis: A prospective cohort study. British J. Derm..

[41] Zhu Y., Hu C., Lu M., Liao S., Marini J.C., Yohrling J., Yeilding N., Davis H.M., Zhou H. (2009). Population pharmacokinetic modeling of ustekinumab, a human monoclonal antibody targeting IL-12 ⁄ 23p40, in patients with moderate to severe plaque psoriasis. J. Clin. Pharmacol..

[42] Lebwohl M., Yeilding N., Szapary P., Wang Y., Li S., Zhu Y., Reich K., Langley R.G., Papp K.A. (2010). Impact of weight on the efficacy and safety of ustekinumab in patients with moderate to severe psoriasis: Rationale for dosing recommendations. J. Am. Acad. Dermatol..

[43] Papp K., Kimball A., Wasfi Y., Chan D., Bissonnette R., Sofen H., Yeilding N., Li S., Szapary P., Gordon K.B. (2012). Long-term efficacy and safety of ustekinumab in patients with moderate to severe psoriasis through 5 years of follow-up: Results from the PHOENIX 1 long-term extension. J. Dermatol..

[44] Papp K., Langley R., Lebwohl M., Krueger G.G., Szapary P., Yeilding N., Guzzo C., Hsu M.-C., Wang Y., Li S. (2008). Efficacy and safety of ustekinumab, a human interleukin-12/23 monoclonal antibody, in patients with psoriasis: 52-week results from a randomised, double-blind, placebo-controlled trial (PHOENIX 2). Lancet.

[45] Young M.S., Horn E.J., Cather J.C. (2011). The ACCEPT study: Ustekinumab versus etanercept in moderate-to-severe psoriasis patients. Expert Rev. Clin. Immunol..

[46] Zweegers J., van den Reek J., van de Kerkhof P., Otero M.E., Kuijpers A.L.A., Koetsier M.I.A., Arnold W.P., Berends M.A.M., Weppner-Parren L., Ossenkoppele P.M. (2016). Body mass index predicts discontinuation due to ineffectiveness and female sex predicts discontinuation due to side effects in patients with psoriasis treated with adalimumab, etanercept or ustekinumab in daily practice: A prospective, comparative, long-term drug-survival study from the BioCAPTURE registry. Br. J. Dermatol..

[47] Summary of Product Characteristics. https://www.ema.europa.eu/en/documents/product-information/stelara-epar-product-information_en.pdf.

[48] Lee J.E., Wang J., Florian J., Wang Y.-M., Kettl D., Marcus K., Woitach A. (2019). Effect of body weight on risk-benefit and dosing regimen recommendation of secukinumab for the treatment of moderate to severe plaque psoriasis. Clin. Pharmacol. Ther..

[49] Di Minno M.N., Peluso R., Iervolino S., Russolillo A., Lupoli R., Scarpa R., CaRRDs Study Group (2014). Weight loss and achievement of minimal disease activity in patients with psoriatic arthritis starting treatment with tumour necrosis factor α blockers. Ann. Rheum. Dis..

[50] Pona A., Haidari W., Kolli S.S., Feldman S.R. (2019). Diet and psoriasis. Dermatol. Online J..

[51] Murray M., Bergstresser P., Adams-Huet B., Cohen J.B. (2009). Relationship of psoriasis severity to obesity using same-gender siblings as controls for obesity. Clin. Exp. Dermatol..

[52] Gisondi P., Del Giglio M., Di Francesco V., Zamboni M., Girolomoni G. (2008). Weight loss improves the response of obese patients with moderate-tosevere chronic plaque psoriasis to low-dose cyclosporine therapy: A randomized, controlled, investigator-blinded clinical trial. Am. J. Clin. Nutr..

[53] Edson-Heredia E., Sterling K., Alatorre C., Carter G.C., Paczkowski R., Zarotsky V., Maeda-Chubachi T. (2014). Heterogeneity of response to biologic treatment: Perspective for psoriasis. J. Investig. Dermatol..

[54] Al-Mutairi N., Nour T. (2014). The effect of weight reduction on treatment outcomes in obese patients with psoriasis on biologic therapy: A randomized controlled prospective trial. Exp. Opin. Biol. Ther..

[55] Bach E., Nielsen R.R., Vendelbo M.H., Møller A.B., Jessen N., Buhl M., Hafstrom T.K., Holm L., Pedersen S.B., Pilegaard H. (2013). Direct effects of TNF-α on local fuel metabolism and cytokine levels in the placebo-controlled, bilaterally infused human leg: Increased insulin sensitivity, increased net protein breakdown, and increased IL-6 release. Diabetes.

[56] Patel H.J., Patel B.M. (2017). TNF-α and cancer cachexia: Molecular insights and clinical implications. Life Sci..

[57] Tzanavari T., Giannogonas P., Karalis K.P. (2010). TNF-alpha and obesity. Curr. Dir. Autoimmun..

